# Long-Term Effect of a Structured Educational Program on Diabetic Foot on Major Adverse Limb and Cardiovascular Events in People with Type 1 Diabetes

**DOI:** 10.3390/jcm14228149

**Published:** 2025-11-17

**Authors:** Núria Alonso-Carril, Maite Valverde, Jordi Anglada, Luis García-Pascual, Maria-José Barahona, Silvia Rodríguez-Rodríguez, Belén Berrocal, Carmen Quirós, Andreu Simó-Servat, Carlos Puig-Jové, Davinia Martínez, Carme Ferré, Antonio J. Amor, Verónica Perea

**Affiliations:** 1 Hospital Universitari Mútua Terrassa, Dr. Robert 5, 08221 Terrassa, Spain; nuriaalonso@mutuaterrassa.es (N.A.-C.); jordi.anglada@gmail.com (J.A.); mjbarahona@mutuaterrassa.es (M.-J.B.); asimo@mutuaterrassa.cat (A.S.-S.); cpuig@mutuaterrassa.cat (C.P.-J.);; 2 Department of Nursing, Rovira i Virgili University, 43002 Tarragona, Spain; 3 Hospital Clínic de Barcelona, Villarroel 170, 08036 Barcelona, Spain; 4 Institut d’Investigacions Biomèdiques August Pi i Sunyer (IDIBAPS), 08036 Barcelona, Spain; 5 Centro de Investigación Biomédica en Red de Diabetes y Enfermedades Metabólicas Asociadas (CIBERDEM), Instituto de Salud Carlos III (ISCIII), 28029 Madrid, Spain

**Keywords:** type 1 diabetes, foot care, structured educational program, cardiovascular events, major limb events

## Abstract

**Background/Objectives**: To evaluate the long-term impact of a structured diabetic foot education program on major adverse limb events (MALEs) and major adverse cardiovascular events (MACEs) in individuals with type 1 diabetes. **Methods**: Retrospective cohort study including 327 adults with type 1 diabetes who participated in a structured diabetic foot education program between 1990 and 2014. The program included 10 h of education focused on diabetes self-management and foot care. Participants were encouraged to attend annual refresher sessions. The primary outcomes were the first occurrence of MALE (major amputation or lower-limb revascularization) and MACE (non-fatal stroke, non-fatal myocardial infarction, or all-cause death). Cox proportional hazards models were used to assess associations between the number of educational programs (EPs) attended (1, 2–3, >3) and outcomes. **Results**: Participants attended a median of two EPs during a median follow-up of 26.3 years. Of the total number of events, 21 were MALEs (crude incidence of 1.9 per 1000 person-years) and 108 were MACEs (incidence of 12.9 per 1000 person-years). Risk of MALE was not associated with the number of EPs attended (*p* = 0.384). In contrast, participants who attended >3 EPs had a significantly lower risk of MACE compared with those attending only one program (HR 0.48, 95% CI 0.24–0.99, *p* = 0.044), although this association was attenuated after adjusting for smoking status. **Conclusions**: Frequent participation in a structured diabetic foot education program was associated with fewer cardiovascular events, but this benefit was diminished by smoking. Integrating tobacco cessation into education programs may enhance their long-term impact.

## 1. Introduction

Diabetic-related foot disease is a chronic complication of diabetes mellitus characterized by peripheral neuropathy, peripheral arterial disease (PAD), infection, ulcers, neuro-osteoarthropathy, gangrene, or amputation [1,2,3,4]. Globally, up to 34% of individuals with diabetes are expected to develop a diabetic-related foot ulcer (DFU) during their lifetime [5,6,7]. These complications are not only a leading cause of disability and hospitalization but are also associated with major adverse cardiovascular events (MACEs) and increased all-cause mortality [8]. Indeed, individuals with a history of DFU or amputation have a significantly higher risk of death, underscoring the systemic impact of this condition beyond the limb itself [7,9].

Diabetes self-management education and support (DSMES) is a critical element of care for all people with diabetes. It supports individuals in acquiring the knowledge, skills, and confidence needed to manage their condition, prevent complications, and improve quality of life [10,11]. Structured foot care education should include instruction on foot hygiene, appropriate footwear, daily self-examination, and prompt recognition of warning signs [4]. While DSMES has been related to improved glycemic control and increased adherence to treatment [12,13,14], its role in preventing diabetic foot complications and mortality has been scarcely assessed [15,16].

Previous studies suggest that structured education, delivered through systematic protocols, can improve knowledge and promote preventive foot care behaviors [17,18]. However, research on their specific effectiveness in preventing DFUs and amputations shows mixed results, with studies primarily focusing on broader populations rather than those with type 1 diabetes. A meta-analysis of five randomized controlled trials (RCTs) showed a relative risk reduction for ulceration (RR 0.66; 95% CI: 0.37–1.19) in favor of structured education, although the results did not reach statistical significance [19]. This limited body of evidence highlights the need for further investigation, particularly in individuals with type 1 diabetes, where recent studies have indicated that the risk of diabetes-related foot disease may be comparable to that observed in type 2 diabetes [20]. Moreover, the long-term impact of such interventions on hard outcomes such as cardiovascular disease and mortality remains largely unknown in this particularly high-risk population of individuals with type 1 diabetes [16].

Filling the gaps in knowledge and practice is essential for implementing strategies to improve long-term outcomes in people with type 1 diabetes. To this end, this study assesses the long-term effectiveness of a structured diabetic foot care education program in reducing major limb events, cardiovascular complications, and mortality, specifically in individuals living with type 1 diabetes.

## 2. Materials and Methods

### 2.1. Study Design and Population

We conducted a retrospective cohort study including individuals with type 1 diabetes aged ≥18 years who participated in a structured diabetic foot education program at a university hospital (Hospital Universitari Mútua Terrassa, Terrassa, Spain) between 1990 and 2014. Participants who moved outside the hospital’s catchment area or lacked documented medical visits during the follow-up period were excluded. The study and data analysis were conducted between December 2024 and January 2025. This manuscript was prepared in accordance with the Strengthening the Reporting of Observational Studies in Epidemiology (STROBE) statement (Supplemental Materials). The study was conducted according to the guidelines of the Declaration of Helsinki and approved by the Ethics Committee of Fundació Assistencial Mutua Terrassa (protocol code P/24-130, date of approval 4 November 2024).

### 2.2. Intervention

The structured diabetic foot education program was implemented at the Diabetes Unit of the Hospital Universitari Mútua Terrassa and delivered by a multidisciplinary team of endocrinologists and diabetes nurse educators. All adults with diabetes attending the Unit were offered the opportunity to participate in the program on a voluntary basis. For the present study, only participants with type 1 diabetes were included.

Each program consisted of 10 h of education, delivered over four weekly sessions (or two condensed sessions when appropriate). The content of the program was developed before the publication of the first International Consensus on the Diabetic Foot [21]. Nonetheless, the curriculum was designed to cover two main areas: (1) general diabetes self-management, following the ABCDES framework (healthy coping, healthy eating, physical activity, medication adherence, glucose monitoring, risk reduction, and problem solving), and (2) prevention of diabetes-related foot complications. Specific foot care content included instruction on appropriate footwear, foot hygiene, skin hydration, nail care, daily foot self-examination, and recognition of warning signs that require medical attention. Participants practiced self-examination techniques, identified personal risk factors, and engaged in hands-on demonstrations to reinforce theoretical knowledge. The educational approach was adapted to each participant’s prior knowledge, learning capacity, and specific needs. Special considerations were made for individuals with visual impairments, limited mobility, or cognitive challenges that could hinder learning or self-care. Each participant underwent a foot examination to assess for deformities, ischemia, and peripheral neuropathy, and to identify specific risk factors. Their footwear and insoles were also reviewed to evaluate wear patterns and determine if modifications were needed to reduce ulcer risk.

Participants were encouraged to attend annual refresher sessions to reinforce knowledge and maintain preventive behaviors.

### 2.3. Routine Diabetes Management and Follow-Up

All participants continued to receive routine clinical follow-up in our Diabetes Unit as part of standard care, independently of their participation in the structured education program. Clinical follow-up visits were scheduled every 3–6 months, depending on the individual’s clinical status and needs, and were performed by endocrinologists and diabetes nurse educators. These visits included clinical assessment, laboratory testing, cardiovascular risk evaluation, and foot examination, according to usual clinical practice. Glycemic targets and treatment strategies were adapted throughout the follow-up period according to the national and international clinical practice guidelines available at each point in time.

### 2.4. Primary Outcomes

The primary outcomes were the first occurrence of a major adverse limb event (MALE) requiring inpatient care during follow-up and the first occurrence of a major cardiovascular event (MACE). MALEs were defined as major amputation (above the forefoot), peripheral endovascular revascularization, or peripheral surgical revascularization of lower limb arteries or the aorta. MACEs included non-fatal stroke, non-fatal myocardial infarction, and all-cause mortality. Outcomes were identified through medical records using International Classification of Diseases (ICD-11) codes [22]. All outcomes were ascertained retrospectively through review of electronic health records up to January 2025.

### 2.5. Covariates

At each educational visit, data on demographics, laboratory parameters, diabetes-related characteristics such as history of foot complications, comorbidities, and physical examination were collected prospectively.

Diabetic kidney disease (DKD) was defined by an albumin-to-creatinine ratio ≥ 30 mg/g and/or an estimated glomerular filtration rate (eGFR) < 60 mL/min/1.73 m^2^. Diabetic retinopathy was diagnosed via non-mydriatic retinography and confirmed by an ophthalmologist. Hypertension was defined as consecutive clinic blood pressure readings ≥ 140/90 mmHg or the use of antihypertensive medications (excluding those used solely for DKD). Dyslipidemia was defined by treatment with lipid-lowering agents such as statins, fibrates, ezetimibe, or resins.

Laboratory parameters were measured in fasting blood and first-morning urine samples. Serum creatinine, glucose, lipid profile (total cholesterol, triglycerides, HDL-cholesterol), and urinary albumin-to-creatinine ratio were assessed using standardized assays. LDL-cholesterol was calculated using the Friedewald formula. eGFR was estimated with the Chronic Kidney Disease–Epidemiology Collaboration (CKD-EPI) equation. HbA1c was measured centrally using high-performance liquid chromatography and expressed in both NGSP and DCCT-aligned units.

### 2.6. Physical Examination

A comprehensive physical exam included assessment of foot health, screening for PAD, and testing for diabetic neuropathy. Loss of protective sensation (LOPS) was defined as the absence of monofilament sensation (using the 10-g monofilament) and at least one other abnormal test (pinprick or vibratory perception using a 128-Hz tuning fork). PAD was defined by absent lower-extremity pulses, prior intervention for peripheral vascular disease, or an ankle-brachial index consistent with current international guidelines [2,23]. Skin lesions were defined as abnormalities in the appearance, texture, or structure of the skin, including lipoid necrosis, fungal infections, hyperhidrosis, fissures, and anhidrosis. Nail lesions included subungual hematoma, onychogryphosis, ingrown toenails, onychomycosis, and dyschromia.

### 2.7. Statistical Analysis

Data are presented as the mean ± standard deviation, median [25th and 75th percentiles] or number (percentage) unless otherwise indicated. Group comparisons were made according to the number of educational sessions attended (1, 2–3, or >3 programs) using ANOVA, Kruskal–Wallis, or Pearson’s chi-squared tests. The Bonferroni test was used as a post hoc analysis to make pairwise comparisons, correcting for multiple analyses.

Cox proportional hazards models were used to estimate the association between the number of educational programs attended and the occurrence of MALE or MACE. The number of programs was modeled as a categorical variable (1, 2–3, or >3). Three models were used: Model 1 adjusted for age and sex; Model 2 included Model 1 plus HbA1c, diabetes duration, hypertension, dyslipidemia, and eGFR; Model 3 included Model 2 plus smoking status at first visit. To address potential selection bias due to high rates of missing eGFR values, multiple imputation for this variable was performed in all aforementioned models. Hazard ratios (HRs) with 95% confidence intervals (CIs) are reported as measures of effect size. The Kaplan–Meier method was used to estimate cumulative incidence curves. To evaluate whether the association between the number of educational programs and the occurrence of MACE differed according to smoking status, we tested an interaction term between smoking (yes/no) and the number of educational programs attended in the Cox proportional hazards models. Statistical significance for interaction was assessed using the likelihood ratio test. A two-sided *p*-value < 0.05 was considered statistically significant. All analyses were performed using STATA 14.0 (College Station, TX, USA).

## 3. Results

### 3.1. Participant Characteristics

A total of 327 subjects with type 1 diabetes were included (Supplemental Figure S1), with a mean age of 42.5 ± 13.7 years, 50.2% were female, a median HbA1c 7.9% (6.9–9.1) (62.8 mmol/mol [51.9–76.0]) and a median duration of diabetes of 14 years (8–22). Most participants had their first visit between 1994 and 2006 (Supplemental Figure S2). Table 1 depicts baseline characteristics at the first educational visit. Notably, foot-related issues were prevalent, with 1.4% of participants having a history of minor amputations below the forefoot, 10% reporting LOPS, and 16% diagnosed with PAD. Upon foot examination, roughly 1 in 10 subjects presented foot lesions, with the most prevalent being skin lesions and callus, and 2.4% had DFUs.

### 3.2. Structured Diabetic Foot Education Program

At the first educational session, 94.9% of participants demonstrated correct hygiene practices, and 90.8% used and fitted their footwear appropriately. However, only 77.8% of individuals with type 1 diabetes practiced adequate foot hydration (e.g., moisturizing). Additionally, 73.4% of participants understood foot care and prevention, while approximately 3% acknowledged knowing what proper foot care involves but admitted to not practicing it.

Supplemental Figure S3 shows the number of programs attended by each participant, with a median of 2 programs per individual. Among those who attended more than one educational program, the median time between sessions was 3 (2–4.4) years. When participants were grouped by the number of programs attended (1, 2–3, or >3 programs), those attending a higher number of sessions were younger, more likely to be female, and had lower rates of hypertension and dyslipidemia (Table 2). No significant between-group differences were observed in terms of diabetes duration, HbA1c levels, diabetes-related complications (including foot complications), or foot lesions at baseline (Table 2).

### 3.3. Events During the Follow-Up

During a median follow-up of 26.3 years (18.3–30.3), n = 8 (2.7%) participants underwent a major amputation, n = 4 (1.3%) had peripheral endovascular revascularization, n = 9 (3.0%) underwent peripheral surgical revascularization, n = 9 (3%) experienced a non-fatal stroke, n = 19 (6.3%) had a non-fatal myocardial infarction, and n = 80 (24.5%) participants died. The causes of death were as follows: n = 17 participants (21.3%) died from non-cardiovascular disease, n = 20 (25.0%) from cardiovascular disease, and n = 43 (53.8%) had an unknown cause of death.

**Figure 1:**
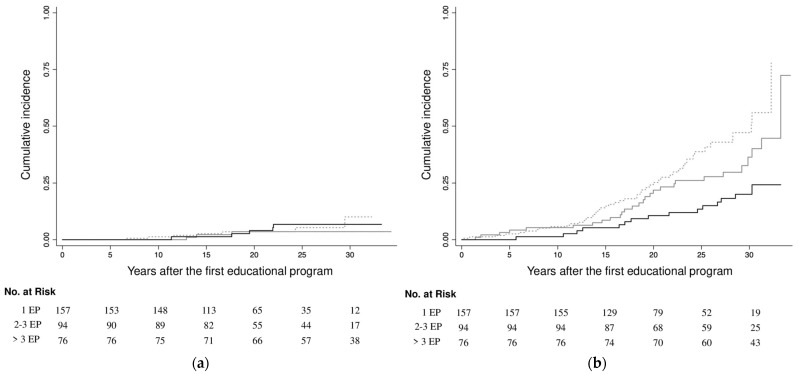
Crude cumulative incidence of the first major limb and cardiovascular event by the number of educational programs (EP) attended. Black solid line: >3 EP; Grey solid line: 2–3 EP; Dashed line: 1 EP. (**a**) First major limb event; (**b**) First major cardiovascular event.

The crude incidence of the first MALE was 1.9 per 1000 person-years, with no significant association between the number of educational programs attended and the occurrence of MALE in either the crude or the fully-adjusted model (>3 educational programs attended: HRcrude 1.01, 95% CI 0.31–3.23, *p* = 0.993; HRadjusted 2.00, 95% CI 0.42–9.60, *p* = 0.384) (Figure 1, Supplemental Table S1).

The crude incidence of the first MACE was 12.9 per 1000 person-years. Participants who attended more than 3 educational programs had a lower risk of MACE (HR 0.31, 95% CI 0.18–0.56, *p* < 0.001) compared to those with 1 education program (Figure 1). This association remained largely unchanged when adjusting for age, sex, HbA1c, diabetes duration, dyslipidemia, hypertension and eGFR (HR 0.48, 95% CI 0.24–0.99, *p* = 0.044). However, when smoking habit was included in the model, the association was blunted (HR 0.56, 95% CI 0.27–1.17, *p* = 0.123) (Table 3). No statistically significant interaction was observed between smoking status and the number of educational programs attended in relation to MACE (interaction *p* = 0.463).

When analyzing the components of MACE, only all-cause mortality was associated with the number of educational programs, following the same pattern as MACE (>3 educational programs attended: HRcrude 0.24, 95% CI 0.12–0.46, *p* < 0.001). This association was lost when smoking habit was included in the adjusted model (HR 0.44, 95% CI 0.19–1.02, *p* = 0.057) (Table 3).

## 4. Discussion

In this study, which included only individuals living with type 1 diabetes, we found that although the incidence of MALEs did not differ significantly, higher adherence to a structured diabetic foot education program was associated with a lower incidence of MACE, particularly all-cause mortality. This inverse association remained significant even after adjusting for multiple confounders, except for smoking status, highlighting the critical role of smoking in the overall prognosis of individuals with type 1 diabetes. To our knowledge, this is among the first pieces of evidence suggesting that a foot-specific DSMES program may also positively impact hard clinical outcomes in the type 1 diabetes population.

Although diabetic-related foot disease, particularly DFUs, is generally considered less common in individuals with type 1 diabetes compared to those with type 2 diabetes [24], this high-risk complication remains significantly prevalent in type 1 diabetes. Recent studies have reported a prevalence of up to 13–14% among individuals living with type 1 diabetes [25], and a 22-year prospective study suggests that the cumulative risk of foot complications is comparable between type 1 diabetes and type 2 diabetes populations [20]. In line with these findings, our cohort—despite its inherent selection bias—also demonstrated a substantial burden of foot-related complications: 16% showed evidence of PAD, 10% had LOPS, and 1.4% had a history of minor lower-limb amputation (Table 1). These data underscore the need for a structured diabetic foot education program tailored to this specific diabetes population. While the majority of participants adhered to recommended foot hygiene (94.9%) and appropriate footwear use (90.8%) during the initial educational session, notable gaps were observed in foot hydration practices and comprehensive understanding of preventive care. Similar challenges in maintaining adherence—particularly regarding regular foot examinations—were also reported in the DAWN2 study [26]. These findings highlight the importance of repeated educational interventions to reinforce and sustain beneficial self-care behaviors.

The effectiveness of structured educational programs in reducing the risk of diabetes-related foot complications remains a subject of ongoing debate. To date, only a limited number of randomized controlled trials with relatively small sample sizes have addressed this issue, yielding conflicting results [27,28,29,30,31]. While pooled analyses suggest that such interventions can improve patients’ knowledge and certain intermediate outcomes, they have not consistently demonstrated a statistically significant reduction in the incidence of DFUs (RR 0.66; 95% CI: 0.37–1.19) [19]. Similarly, in our study, we did not observe any significant association between the number of educational program sessions attended and the incidence of MALE. Whether this lack of association is attributable to the low number of limb events observed in our study (n = 21)—which could, in turn, reflect the effectiveness of the specific educational program—or to the selection of an overall low-risk population for limb complications remains to be determined. Notably, according to the 2023 IWGDF classification guidelines [4], most participants in our cohort would be categorized as having very low or low risk for diabetic foot complications, which may have limited the statistical power to detect significant associations. Furthermore, given the scarcity of prior evidence specifically focused on individuals with type 1 diabetes, a population-specific effect cannot be ruled out. Interestingly, randomized clinical trials that exclusively enrolled individuals with type 2 diabetes have reported positive outcomes [29,30,31], whereas those including both type 1 diabetes and type 2 diabetes populations have yielded null results [27,28]. These findings suggest that the effectiveness of educational programs targeting foot-related complications may differ by diabetes type. Further research is therefore warranted to determine whether such interventions should be specifically tailored to the unique needs of the type 1 diabetes population.

Diabetes-related foot disease is a well-established prognostic marker in individuals living with diabetes [7]. Several studies have reported that the 5-year mortality rate among people with diabetes who develop diabetic foot ulcers (DFUs) can reach up to 40%, and this figure increases to over 60% in those who undergo lower-limb amputation [32,33]. Interestingly, a recent population-based study including over 71,000 individuals with newly diagnosed DFU (both with type 1 and type 2 diabetes) reported a 52-week mortality rate exceeding 14%. Although mortality was lower among individuals with type 1 diabetes, the 1-year mortality still approached 10%, underscoring the severity of DFUs across diabetes types [9]. In this context, our findings showing that adherence to a structured foot-specific education program may be associated with a lower incidence of MACE, particularly driven by a reduction in all-cause mortality, are especially noteworthy. Although previous meta-analyses have demonstrated that DSMES is associated with reduced mortality in individuals with type 2 diabetes [16], this is, to our knowledge, the first evidence suggesting that a foot-focused educational intervention may also confer a protective effect, particularly among individuals with type 1 diabetes—a population that has been largely underrepresented in prior studies. Furthermore, our results are compelling, as previous data have shown that receiving at least 10 h of education and attending repeated appointments may be associated with improved outcomes [16,34]. Strategies to improve adherence, such as telecare, personalized reminders, and motivational interventions, merit exploration to increase the reach and impact of such programs [14].

Smoking is one of the most detrimental behavioral risk factors in individuals living with type 1 diabetes. It has been strongly associated with an increased risk of diabetes-related foot complications [24] and cardiovascular disease [35,36]. In fact, among individuals with adult-onset type 1 diabetes, smoking habit represents the leading population-attributable risk factor for overall mortality [37]. The impact of smoking is also evident in our study, as the inverse association between participation in the foot-specific education program and the incidence of MACE and all-cause mortality was attenuated after adjusting for baseline smoking status (Table 3). These findings highlight the critical role of smoking cessation in mitigating cardiovascular and mortality risk, also among the type 1 diabetes population [38]. Addressing tobacco use within educational programs could amplify their benefits, considering the combined impact of smoking and hyperglycemia on vascular complications [36,37].

This study presents several strengths and limitations. Its principal strength lies in the extensive 26-year follow-up, which offers a robust dataset for assessing long-term outcomes in individuals with type 1 diabetes. Moreover, it focuses on a population that has been underrepresented in the literature to date. Most existing evidence on diabetic foot complications stems from studies involving individuals with type 2 diabetes. Therefore, our findings may serve as a valuable starting point for developing tailored prevention and management programs specifically designed for people with type 1 diabetes. Nonetheless, several limitations must be acknowledged. First, as an observational study, the findings are susceptible to potential confounding factors that may affect their interpretation. The lack of a control group and the non-randomized design further constrain the ability to draw causal inferences. Second, the study cohort was derived from a single center, which may limit the external validity and generalizability of the results to broader populations. Third, missing data represent a potential limitation. However, multiple imputation techniques were applied to address this issue, which are widely recognized as valid and robust methods for handling incomplete datasets in observational research [39]. Fourth, socioeconomic status, which has been shown to strongly influence glycemic control, foot ulcer incidence and amputation risk [3], was not systematically available in our dataset and could therefore not be included in the analyses. This should be considered when interpreting the results. Finally, data regarding changes in knowledge and behavioral habits following the specific educational intervention were not collected. Consequently, it is not possible to determine with certainty whether the observed improvements in outcomes were exclusively mediated by the acquisition of healthier habits.

## 5. Conclusions

In conclusion, our findings suggest that improving adherence to structured diabetic foot education programs, although not associated with a reduction in MALEs, may confer benefits on other clinically relevant outcomes, particularly MACEs, including all-cause mortality. The observed reduction in MACEs among participants who attended multiple sessions underscores the potential value of sustained engagement and longitudinal follow-up. However, the attenuation of this association after adjustment for smoking status highlights the critical need to incorporate comprehensive smoking cessation strategies into diabetes education programs, given its role as a key modifiable risk factor for both cardiovascular and limb-related complications. Table S1. Risk of major adverse limb events according to the number of educational programs attended.

## Figures and Tables

**Table 1:** Baseline characteristics of participants at the first educational session.

			N = 327
Demographic characteristics	Age (years)		42.5 ± 13.7
	Sex (female)		164 (50.2)
	Higher education		30/276 (10.9)
	European descendent		327 (100)
	Active smoking habit		91/307 (29.7)
	Diabetes duration (years)		14 (8–22)
	Comorbidities and cardioprotective drugs		
	Hypertension		48/306 (15.7)
	Dyslipidemia		59/280 (21.1)
	Use of antiplatelets		21/288 (7.3)
	Diabetes-related complications		
	Retinopathy		91/233 (39.1)
	DKD		16/112 (14.3)
	Neuropathy		39/285 (13.7)
	Myocardial infarction		10/278 (3.6)
	Stroke		3/273 (1.1)
	Foot complications history		
	Major amputation (above the forefoot)		0/295
	Minor amputation (below the forefoot)		4/295 (1.4)
	Loss of protective sensation		30/299 (10.0)
	Peripheral artery disease		50/312 (16.0)
Physical examination	Body mass index	N	286
	Kg/m^2^		26.0 ± 17.3
	Systolic blood pressure (mmHg)		130 ± 19
	Diastolic blood pressure (mmHg)		76 ± 13
	Foot lesions		
	Hammertoes		5/280 (1.7)
	Bunions		9/299 (3.0)
	Callus		33/299 (11.0)
	Diabetic foot ulcers		7/296 (2.4)
	Skin lesions		31/293 (10.6)
	Nail lesions		21/292 (7.2)
Laboratory measures	HbA1c	N	283
	%		7.9 (6.9–9.1)
	mmol/mol		62.8 (51.9–76.0)
	Total cholesterol	N	270
	mg/dL		187 (165–213)
	Triglycerides	N	264
	mg/dL		87 (64–123.5)
	HDL-cholesterol	N	191
	mg/dL		51 (42–63)
	LDL-cholesterol	N	190
	mg/dL		114.2 (97–139.6)
	Serum creatinine	N	151
	mg/dL		0.98 (0.9–1.1)
	eGFR(CKD-EPI)	N	151
	mL/min/1.73 m^2^		91 (78.9–101)

Results are given as n (%), n/N (%) in case of missing data, mean ± SD for normal distributions or median (IQR) for non-normal distributions. Abbreviations: DKD, diabetic kidney disease; eGFR, estimated glomerular filtration rate.

**Table 2:** Baseline characteristics of participants at the first educational session according to the number of programs attended.

		1 EP (n = 157)	2–3 EP (n = 94)	>3 EP (n = 76)	*p*
Demographic characteristics					
Age (years)		44.9 ± 14.6	41.1 ± 13.1 †	39.3 ± 11.5 †	0.007
Sex (female)		69 (44.2)	47 (50)	47 (61.8)	0.042
Higher education		9/116 (7.8)	11/88 (12.5)	10/72 (13.9)	0.354
Active smoking habit		46/142 (32.4)	28/92 (30.4)	17/73 (23.3)	0.376
Diabetes duration (years)		13 (7–22)	13.5 (9–24)	15 (8.5–23)	0.619
Comorbidities and cardioprotective treatment					
Hypertension		32/141 (22.7)	10/90 (11.1)	7/75 (9.3) †	0.012
Dyslipidemia		40/118 (33.9)	18/88 (20.5)	1/74 (1.35) †, ‡	<0.001
Use of antiplatelets		15/130 (11.5)	5/84 (6.0)	1/74 (1.4) †	0.023
Diabetes-related complications					
Retinopathy		37/109 (33.9)	33/74 (44.6)	21/50 (42.0)	0.312
DKD		5/56 (8.9)	3/25 (12.0)	8/31 (25.8)	0.092
Neuropathy		17/134 (12.7)	13/86 (15.1)	9/65 (13.9)	0.876
Myocardial infarction		4/133 (3.0)	3/81 (3.7)	3/64 (4.7)	0.786
Stroke		2/131 (1.5)	1/81 (1.2)	0	0.769
Foot complications history					
Major amputation (above the forefoot)		0	0	0	-
Minor amputation (below the forefoot)		2/144 (1.4)	1/87 (1.2)	1/64 (1.6)	0.976
Loss of protective sensation		16/147 (12.9)	7/85 (8.2)	4/67 (6.0)	0.236
Peripheral artery disease		23/151 (15.2)	13/90 (14.4)	14/71 (19.7)	0.620
Physical examination					
Body mass index	N	122	91	73	
Kg/m^2^		27.5 ± 26.2	25.0 ± 3.8	24.8 ± 3.8	0.449
Systolic blood pressure (mmHg)		129 ± 18	130 ± 19	132 ± 18	0.493
Diastolic blood pressure (mmHg)		75 ± 10	76 ± 13	78 ± 15	0.283
Foot lesions					
Hammertoes		2/130 (1.5)	3/83 (3.6)	0	0.241
Bunions		2/145 (1.4)	5/87 (5.8)	2/67 (3.0)	0.169
Callus		13/145 (9.0)	12/87 (13.8)	8/67 (11.9)	0.506
Diabetic foot ulcers		2/144 (1.4)	2/87 (2.3)	3/65 (4.6)	0.364
Skin lesions		9/143 (6.3)	12/86 (14.0)	10/64 (15.6)	0.063
Nail lesions		12/142 (8.5)	3/87 (3.5)	6/63 (9.5)	0.262
Laboratory measures					
HbA1c	N	134	80	69	0.057
%		8.1 (7.3–9.3)	7.8 (6.6–8.8)	7.6 (6.5–8.9)	
mmol/mol		65.0 (56.3–78.1)	61.7 (48.6–72.1)	59.6 (47.5–73.8)	
Total cholesterol	N	123	74	73	0.556
mg/dL		186 (158–213)	184 (165–225)	191 (174–207)	
Triglycerides	N	120	73	71	0.014
mg/dL		84.5 (70.5–129.5)	79 (61–108)	83 (63–101) †	
Cholesterol-HDL	N	88	50	53	0.137
mg/dL		47.5 (40–61)	55.5 (44–68)	52 (44–64)	
Cholesterol-LDL	N	87	50	53	0.582
mg/dL		114.2 (94.8–135.6)	115.7 (87.4–148.4)	112.8 (105–140.2)	
Serum creatinine	N	71	37	43	0.734
mg/dL		0.92 (0.9–1.1)	1 (0.9–1.1)	1 (0.9–1.1)	
eGFR(CKD-EPI)	N	71	37	43	0.9201
mL/min/1.73 m^2^		93.2 (77.0–105.3)	91.9 (80.5–100.7)	90.0 (82.0–96.7)	

Results are given as n (%), n/N (%) in case of missing data, mean ± SD for normal distributions or median (IQR) for non-normal distributions. † *p* < 0.05 vs. 1 educational program. ‡ *p* < 0.05 vs. 2–3 educational programs. Abbreviations: DKD, diabetic kidney disease; EP, educational program; eGFR, estimated glomerular filtration rate.

**Table 3:** Risk of major adverse cardiovascular events according to the number of educational programs attended.

Outcome	EP	n/N (%)	Crude Model HR (95% CI)	Adjusted Model 1 HR (95% CI)	Adjusted Model 2 HR (95% CI)	Adjusted Model 3 HR (95% CI)
First MACE	1	49/157 (31.2)	1 (reference)	1 (reference)	1 (reference)	1 (reference)
	2–3	28/94 (29.8)	0.67 (0.41–1.07)	1.21 (0.73–2.01)	1.02 (0.58–1.82)	1.10 (0.60–2.03)
	>3	16/76 (21.1)	0.31 (0.18–0.56) *	0.56 (0.30–1.03)	0.48 (0.24–0.99) *	0.56 (0.27–1.17)
Components of MACE:						
Non-fatal stroke	1	4/141 (2.8)	1 (reference)	1 (reference)	1 (reference)	1 (reference)
	2–3	3/88 (3.4)	1.09 (0.24–4.88)	1.46 (0.31–6.90)	1.45 (0.17–14.41)	1.49 (0.17–13.04)
	>3	2/73 (2.7)	0.80 (0.14–4.37)	1.12 (0.19–6.77)	1.75 (0.17–17.57)	2.26 (0.20–25.55)
Non-fatal myocardial infarction	1	6/141 (4.3)	1 (reference)	1 (reference)	1 (reference)	1 (reference)
	2–3	6/89 (6.7)	1.31 (0.42–4.09)	1.55 (0.48–4.96)	0.83 (0.23–2.87)	0.75 (0.21–2.62)
	>3	6/73 (8.2)	1.27 (0.40–4.03)	1.56 (0.47–5.21)	0.88 (0.27–2.97)	0.84 (0.24–2.85)
Death from any cause	1	44/157 (28.0)	1 (reference)	1 (reference)	1 (reference)	1 (reference)
	2–3	24/93 (25.8)	0.59 (0.36–0.98) *	1.06 (0.62–1.80)	0.95 (0.50–1.80)	1.04 (0.54–2.02)
	>3	12/76 (15.8)	0.24 (0.12–0.46) *	0.45 (0.22–0.89) *	0.36 (0.16–0.83) *	0.44 (0.19–1.02)

Data are n/N(%) and Hazard Ratio (HR) and 95% confidence interval (CI). * *p* < 0.05. Model 1 included age and sex. Model 2 included model 1 plus HbA1c at the first educational program, diabetes duration, hypertension, dyslipidemia and estimated glomerular filtration rate. Model 3 included model 2 plus smoking habit. Abbreviations: EP, educational programs; MACE, major adverse cardiovascular event.

## Data Availability

The datasets used and analysed during the current study are available from the corresponding authors upon reasonable request.

## Supplementary Materials

The following supporting information can be downloaded at https://www.mdpi.com/article/10.3390/jcm14228149/s1. Figure S1. Flowchart of the cohort of people with type 1 diabetes who participated in the structured diabetic foot education program, including detailed information on the population excluded from the study. Figure S2. Distribution of the number of subjects included per year. Figure S3. Distribution of the number of programs attended by each individual. Table S1. Risk of major adverse limb events according to the number of educational programs attended.

## Abbreviations

The following abbreviations are used in this manuscript:

CKD-EPI	Chronic Kidney Disease–Epidemiology Collaboration DFU: diabetic-related foot ulcer
DFU	Diabetic kidney disease
DKD	Diabetic kidney disease
DSMES	Diabetes self-management education and support
eGFR	Estimated glomerular filtration rate
EPs	Educational programs
LOPS	Loss of protective sensation
MACE	Major adverse cardiovascular event
MALE	Major adverse limb event
RCTs	Randomized controlled trials
PAD	Peripheral arterial disease
STROBE	Strengthening the Reporting of Observational Studies in Epidemiology
